# Efficacy of a blended learning programme in enhancing the communication skill competence and self-efficacy of nursing students in conducting clinical handovers: a randomised controlled trial

**DOI:** 10.1186/s12909-022-03361-3

**Published:** 2022-04-13

**Authors:** Jessie Yuk Seng Chung, William Ho Cheung Li, Ankie Tan Cheung, Laurie Long Kwan Ho, Joyce Oi Kwan Chung

**Affiliations:** 1 School of Nursing, Tung Wah College, Hong Kong, China; 2 The Nethersole School of Nursing, The Chinese University of Hong Kong, 8/F, Esther Lee Building Sha Tin, Hong Kong, China; 3 School of Nursing, The Hong Kong Polytechnic University, Hong Kong, China

**Keywords:** Blended learning, Online module, Clinical handover, Communication skill competence, Self-efficacy, Nursing students

## Abstract

**Background:**

A clinical handover is an essential nursing practice that ensures patient safety. However, most newly graduated nurses struggle to conduct clinical handovers as they lack sufficient communication skill competence and self-efficacy in this practice. This study aimed to examine the efficacy of a blended learning programme on the communication skill competence and self-efficacy of final-year nursing students in conducting clinical handovers.

**Methods:**

A randomised controlled design was used. A convenience sample of 96 final-year baccalaureate nursing students at a local university. Data were collected in 2020. Participants were randomly assigned to either an experimental group (*n* = 50) that received a blended learning programme with face-to-face training and an online module on handover practice, or a waitlist control group (*n* = 46) that received only face-to-face handover training during the study period and an online module immediately after the completion of data collection. The primary outcome was the communication skill competence and the secondary outcome was the self-efficacy of the participants in conducting clinical handovers. An analysis of covariance was used to examine the between-subjects effects on self-efficacy and communication skill competence in conducting clinical handovers after controlling for the significantly correlated variables. A paired sample t-test was used to determine the within-subjects effects on self-efficacy.

**Results:**

The participants in the experimental group had significantly higher communication skill competence (*p* < 0.001) than those in the waitlist control group. Although both groups showed a significant improvement in self-efficacy, the mean scores of the experimental group were higher than those of the waitlist control group (*p* < 0.001).

**Conclusions:**

This study demonstrated the efficacy of a blended learning approach in improving the communication skill competence and self-efficacy of final-year nursing students in conducting clinical handovers. Nurse educators should incorporate a blended learning approach into the nursing curriculum to optimise the content of training programmes for teaching nursing students in conducting clinical handovers.

**Trial registration:**

The study protocol was registered in the Registration ClinicalTrials.gov (NCT05150067; retrospective registration; date of registration 08/12/2021).

**Supplementary Information:**

The online version contains supplementary material available at 10.1186/s12909-022-03361-3.

## Background

A clinical handover is an essential nursing practice to ensure patient safety by conveying relevant patient care information and facilitating countinity of patient care from one healthcare provider to another [1, 2]. To enable the provision of continuous patient care, the handover information must be clear, concise and systematic [3]. Communication and information that are unclear and unsystematic often impede the clarity of ideas, lead to the omission of important patient information and delay medical treatment, ultimately affecting patient safety [4–6]. Previous studies conducted in the United States, United Kingdom and Korea have shown that ineffective clinical handovers can impose huge financial burdens on the healthcare system [7–9].

A review of the literature revealed that nursing students and newly graduated nurses often struggle with clinical handovers due to a lack of communication skill competence and self-efficacy in performing this practice [10–12], especially delivering clinical handover, which is a source of frustration for the newly graduated nurses [13, 14]. A recent qualitative study examined the experiences of newly graduated nurses in Hong Kong in performing clinical handovers and found that most of them perceived barriers to handover, such as inadequate professional judgement, poor ability to synthesise important information and unsystematic reporting [15]. Moreover, many of the nurses claimed in interviews that they were not adequately prepared for clinical handovers during their undergraduate course [15]. It is well documented that education and practice play essential roles in enhancing effective communication during handover [16, 17]. However, teaching clinical handover practice to nursing students is often overlooked and not given enough importance [15]. The current nursing curriculum in the local institutions also lack a well-structured and comprehensive training programme for teaching the communication skills required for efficient handover [15]. Therefore, a revision of the current nursing curriculum to include a customised clinical handover training programme that maximises the students’ knowledge and skills in communication and thus builds their competence and self-efficacy is of paramount importance [18].

Blended learning programmes (BLPs) are commonly used as constructivist pedagogical approaches in nursing education [19]. They involve both face-to-face components and online engagement [20]. The online learning components are usually flexible, inexpensive and can be easily adapted to individual needs and completed at any time and location of choice. This allows learners to be in full control of the learning pace [21]. The online learning modules also provide multiple learning opportunities to the participants, which they can use to practice the skills they learn until they attain mastery. A recent study suggested that blended learning approaches can also solve the issue of large class [22] as it allows instructors to address a large number of learners at the same time and provide adequate opportunities for the learners to practice their skills.

The use of BLPs has been proven successfully in nursing education in terms of teaching theoretical courses and psychomotor skills in nursing education, as well as enhancing the knowledge [23, 24], self-efficacy [23], motivation [25], attitudes [26] and perceived competence of students [27]. Despite this, no studies that examine the efficacy of BLPs in improving nursing students’ communication skill competence and self-efficacy in conducting clinical handovers have been identified in PubMed, in the Cochrane Library or on clinical trial registries (ClinicalTrials.gov and ISRCTN). In this study, we aimed to test the efficacy of BLP on nursing students’ communication skill competence and self-efficacy in performing clinical handovers.

### Theoretical framework

The blended clinical handover training programme used in this study was guided by the self-efficacy theory [28]. Bandura defines self-efficacy as an individual’s belief in his/her own abilities to achieve a certain level of performance in solving a difficult task. The theory identifies four sources of self-efficacy: mastery experiences, vicarious experiences, verbal persuasion and physiological arousal [28]. A direct experience of mastery is the most powerful means of increasing self-efficacy. Vicarious experiences are achieved by observing one’s peers; observing successful peers helps students develop the belief that they too can succeed. Verbal persuasion, such as teacher feedback, encouragement and other external reinforcements, can positively strengthen one’s self-efficacy. Physiological arousal can manifest as negative emotions such as anxiety, high levels of stress and self-doubt, which can negatively impact one’s perception of self-efficacy.

The clinical handover training programme was developed based on the theory that included learning from own experience, learning from peers, feedback on their performance, and positive reinforcement to reduce their anxiety. Therefore, the online module provided some scenario-based exercises that allowed students to use the learned handover technique in conducting handover reporting. After each exercise, the best performed clinical handover record of each exercise was upload anonymously to the module as a model. Students could listen to it and learn how to perform the reporting in a better way. Teacher also provided individual feedback on students’ performance. The areas that the student had done well were reinforced and constructive suggestions on the areas they needed to improve were provided. Students were also encouraged to use the online forum to exchange their opinions. All of the above learning experiences will strengthen the students’ self-efficacy and consequently enhance their competence in conducting clinical handovers.

## Methods

### Study design and setting

A single-centre, two-arm, parallel design randomised control trial (RCT) with a waitlist control group was conducted in a university between 4 May 2020 and 8 June 2020. Students in the waitlist control group would receive the same intervention as students in the intervention group after the completion of the study. Using the waitlist control group was to ensure that no students were disadvantaged as a result of different group assignment. The study strictly followed the Consolidated Standards of Reporting Trials (CONSORT) 2010 guidelines.

### Participants and sample size

Convenience sampling was used to recruit final-year baccalaureate nursing students. They were eligible to participate if they: a) were Hong Kong residents who could speak Cantonese and read Chinese and English; b) were aged at least 18 years; and c) had not previously enrolled in a clinical handover training programme.

G*Power 3 was used to estimate the sample size [29]. With reference to a previous study on handover training for medical students [30], the effect size for the proposed intervention was 0.60 (Cohen’s d; 0.2, 0.5, 0.8 are typically interpreted as small, moderate and large effect sizes respectively) [31]. The research team reached a consensus that such changes constitute a minimally importance difference and thus warrant a change in nursing curriculum. To detect significant differences between the two groups (two tails) with an effect size of 0.60, a level of significance of 5% and power of 0.8, a minimum sample size of 90 participants was required. Considering a potential retention rate of 95%, at least 95 participants were required.

### Randomisation and concealment

The participants were randomly assigned to either the intervention group or waitlist control group. Randomisation was performed by a research team member who opened a serially labelled, opaque, sealed envelope that contained a card indicating a randomly allocated group. The random numbers used for group assignment were generated before participant recruitment by another research team member using an online software and stored in a personal computer to ensure no one can retrieve the sequence.

### Interventions

The training programme aimed to improve the communication skill competence and self-efficacy of nursing students in conducting clinical handovers. The content of the training programme was designed to match the learning needs of newly graduated nurses regarding clinical handover. Therefore, it covered the identification of key information for handover, synthesis of nursing assessments and important patient needs, and systematic handover reporting [15]. Thus, a well-structured BLP for handover practice with a face-to-face training workshop and an online module was developed based on Madeline Hunter’s direst instruction design, which is highly structured and repetitive [32] (please see Supplement 1).

The face-to-face training workshop aimed to provide knowledge of clinical handovers and introduce the Situation, Background, Assessment, Recommendation (SBAR) technique. The SBAR technique was included in the training workshop to ensure efficient communication among the nursing students [25, 26]. It serves as a checklist for nursing communication and enhances their confidence when conversing with other healthcare professionals [25–27]. Previous studies have found that successful application of the SBAR technique could reduce patient risk [33] and increase patient and staff satisfaction in high-risk environments such as intensive care units, emergency rooms and operating rooms [34]. The face-to-face training workshop was delivered through a PowerPoint presentation conducted by an experienced nurse educator who is familiar with clinical handover and the SBAR tool. The PowerPoint slides were reviewed by an expert panel composed of four members including two nurse educators and two registered nurses. The nurse educators reviewed and gave comments on the content and the flow of the face-to-face training workshop. For all sessions, including the PowerPoint presentation, the demonstration of handover practice, and group discussion, were two-hour-long.Within 2 weeks of completing the face-to-face training workshop, the participants in the experimental group were invited to access the online module. The online module aimed to provide ample practice to master the technique, sufficient opportunities to learn from the outstanding reports of their peers and thereby gain vicarious experiences, and enough constructive feedback on their performance to achieve verbal persuasion and physiological arousal. It was created on Google Classroom, which was provided by the collaboration of the university and Google Suite for Education. The case scenarios and exercises had been uploaded to the platform, and the exercises’ cut-off dates were set before the training started. Participants in the experimental group had been invited to use the online module after the training workshop. There were four tasks in the online module, the participants had to complete them one by one and send the audio record of their handover report to the instructor before the due date to obtain feedback, and then they could proceed to the next task. The whole process of the study was monitored by the research office and the school.

A briefing session was given to all participants at the beginning of the study to highlight the importance of giving clinical handover in order to increase their awareness to the practice and adherence of the online module. During the study, because the online learning was delivered through the online platform, participants had to use their individual accounts to log in to the module; thus, the instructor could monitor whether they have a login. Moreover, the participants were requested to complete the four online exercises one by one and upload the audio record on the module. Therefore, the instructor could follow their learning progress and remind them if they forget to submit the recording. Finally, individual comments provided by the instructor were constructive and valuable to the participants that improve their engagement in the online module.

The participants in the waitlist control group received the same face-to-face training workshop as the experimental group. However, these participants were invited to access the online module only after data collection was completed.

### Measures and instruments

The primary outcome was the participants’ communication skill competence in conducting clinical handovers, which was assessed using the SBAR Communication and Communication Clarity tool. The assessment had total 23 items that were rated on a Likert scale. SBAR Communication contained 12 items that were rated on a 3-point Likert scale (0 = not perform, 1 = lacking, 2 = reasonable) with a total score range of 0 to 24 points. The scale was used to examine students’ skill performance of adopting SBAR technique in clinical handover. The assessor based on the completeness of the content in different areas, such as medical history, assessment, treatment, and recommendation, to rate from 0 to 2. Higher scores correspond to better performance on using the SBAR technique in clinical handover. Cronbach’s α for the scale was 0.58 in Yu and Kang’s study [35] and 0.72 in this study.

Communication Clarity contained 11 items that were rated on a 5-point Likert scale (1 = strongly disagree, 2 = disagree, 3 = average, 4 = agree, 5 = strongly agree) with a total score range of 11 to 55 points. The scale aimed to assess whether the participants are able to identify important information and transfer it accurately and understandably. For example, the assessor evaluate whether the participant able to present the verbal handover report in a logical flow, provide a clear summary of an important problem, and present the client’s clinical information accurately. Higher scores indicated more clearance of their clinical handover report. Cronbach’s α for the scale was 0.84 in Yu and Kang’s study [35] and 0.90 in this study. The tool was reviewed for content validity by an expert panel that consisted of clinical nurses and nursing faculty. The I-CVI of all the items in SBAR Communication and Communication Clarity were higher than 0.8, and the S-CVI/Ave of the SBAR Communication and Communication Clarity were 0.96 and 1, respectively.

The case scenario developed for skill assessment was reviewed by the expert panel and finalised after multiple reviews and modifications. The expert panel commented that the content and level of difficulty of the case scenario were appropriate for the assessment of communication skill competence. The participants were requested to conduct a clinical handover based on the case scenario, audio record the report and submit it to the online module. All of the handover reports were audio recorded anonymously and saved using an assigned number code to reduce impression-induced biases. To enhance the reliability of the assessment and reduce the discrepancy in ratings, we used only a single assessor [36].The secondary outcome was participants’ self-efficacy in conducting clinical handovers. Visual analogue scale (VAS) is a valid, sensitive and reliable method for measuring subjective feelings of individuals with low distortion and bias [37], which was used to assess participants’ self-efficacy in conducting clinical handovers. Participants were asked: “Indicate how confident you believe yourself to be in conducting clinical handovers.” The participants were then requested to drag a sliding bar from 0 to 100 on the online survey platform to indicate their level of self-efficacy (0 = Not at all confident, 100 = Extremely confident). The validity of the VAS as a measure of self-efficacy has also been proven by Turner, van de Leemput, Draaisma, Oosterveld and ten Cate [38]. It has been adopted by various studies to assess the self-efficacy and communication skills of nursing students [39] and the level of confidence of nurses in the truth of the information transmitted during handover [40].

### Data collection

After obtaining written informed consent, the participants’ baseline characteristics, such as age, sex and previous experience of observing and performing clinical handovers, and their baseline self-efficacy levels in conducting clinical handovers were collected by a research assistant who was blinded to the group assignment. The participants’ levels of self-efficacy and communication skill competence in conducting clinical handovers were assessed 2 weeks after they received the intervention.

### Data analysis

Data analysis was performed using SPSS for Windows v26.0. Descriptive statistics were used to calculate the mean scores and standard deviations for each scale. The baseline characteristics and research outcomes were compared between the groups using independent samples *t*-test for parametric variables, chi-squared and Fisher’s exact tests for categorical variables and Mann-Whitney U test for continuous variables. The Pearson correlation test and Spearman’s correlation were used to analyse the inter-variable correlations. An analysis of covariance (ANCOVA) was used to examine the between-subjects effects on self-efficacy and communication skill competence in conducting clinical handovers after controlling for the significantly correlated variables. A paired sample *t*-test was used to determine the within-subjects effects on self-efficacy.

### Ethical considerations

This study was approved by the Institutional Review Board of the University of Hong Kong (reference, UW 19–622). An electronic poster with information about the study was sent to the target population. Interested nursing students could contact the study team for enrolment. Nursing students were told that their participation was entirely voluntary and that they had the right to withdraw from the study at any point of time. They were also assured of the confidentiality of their data. An information sheet was provided to all of the participants and written informed consent was obtained.

## Results

### Recruitment and randomisation

**Fig. 1:**
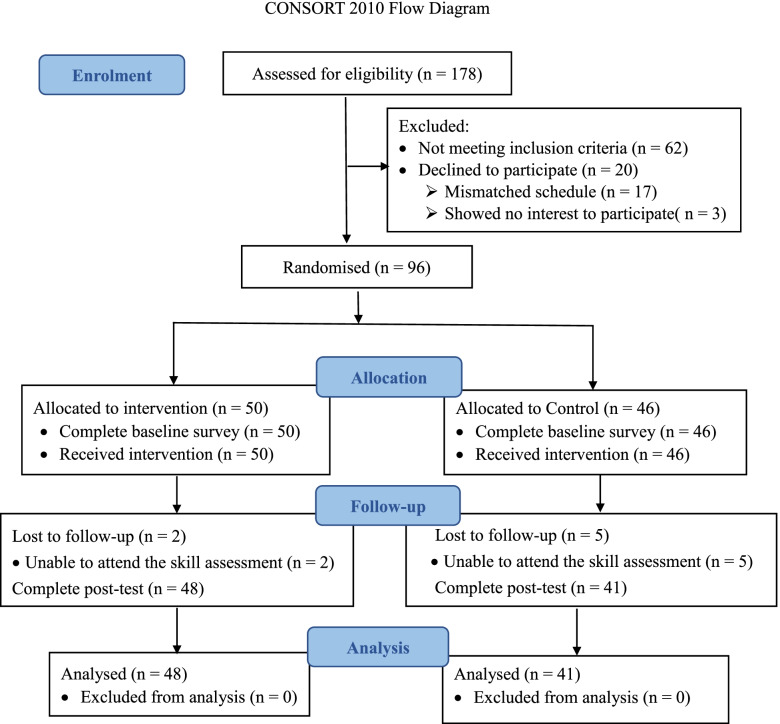
Participant flow from recruitment to post-intervention follow-up

We approached and assessed 178 final-year nursing students from the academic year of 2019 for eligibility. Ninety-six students were enrolled in the study and randomly assigned to either the experimental group or the waitlist control group. Two students in the experimental group and five students in the control group were unable to complete the skill assessment due to physical illnesses. Finally, 89 students completed the study (experimental group: *n* = 48; control group: *n* = 41) (Fig. 1).

### Baseline characteristics of the participants

Table 1 outlines the baseline characteristics of both groups. All of the participants were Chinese, and most of them were female (*n* = 82, 85.4%). The mean (standard deviation) ages of the participants in the experimental group and control group were 23.5 (1.39) years and 23.4 (0.94) years, respectively. There were no statistically significant differences in the demographic or baseline data between those who completed the study and those who dropped out, and between the experimental and control groups.

**Table 1:** Comparison of the demographic and baseline characteristics between the participants in experimental and control groups

	Experimental (***n*** = 48)	Control (***n*** = 41)		
	n (%)	n (%)	***X***^**2**^	***P***
Gender^**a**^			0.48	0.49
Male	7 (14.6)	4 (9.8)		
Female	41 (85.4)	37 (90.2)		
			***Z***	***P***
Previous observe CH^b^			−0.94	0.35
None	1 (2.1)	0 (0.0)		
1–5	10 (20.8)	5 (12.2)		
6–10	8 (16.7)	9 (22.0)		
11–15	5 (10.4)	7 (17.1)		
16–20	7 (14.6)	1 (2.4)		
> 20	17 (35.4)	19 (46.3)		
Previous perform CH^b^			−0.28	0.78
None	22 (45.8)	16 (39.0)		
1–5	23 (47.9)	25 (61.0)		
6–10	3 (6.3)	0 (0.0)		
	**M (SD)**		***T***	***P***
Age^c^	23.50 (1.41)	23.34 (0.97)	−0.61	0.55
Baseline self-efficacy^c^	25.60 (12.95)	27.12 (13.21)	0.55	0.59

Note. *CH* clinical handover

a Chi Square test

^b^ Mann-Whitney U test

^c^ Independent samples *t*-test

Statistical significance was determined at *p* value < 0.05

### Within-subjects effects

Table 2 shows the mean self-efficacy scores of both groups at baseline and after the intervention. We found a statistically significant improvement in the self-efficacy scores of both experimental group, t(47) = 18.70, *p* < 0.001, η^2^ = 0.88, and control group, t(40) = 16.90, *p* < 0.001, η^2^ = 0.88, at 2 weeks after the intervention, regardless of the intervention received.

**Table 2:** Within-Subjects Effects of the Clinical Handover Training Course on the Self-Efficacy

	Baseline self-efficacy Mean (SD)	Posttest self-efficacy Mean (SD)	t	P (2-tailed)	Eta Squared
Control (*N* = 41)	27.12 (13.21)	51.54 (12.10)	t(40) = 16.90	< 0.001	0.877
Experimental (*N* = 48)	25.60 (12.95)	60.94 (15.11)	t(47) = 18.70	< 0.001	0.882

95% confidence interval

### Inter-variable correlation matrix

The participants’ previous experience of observing and performing clinical handovers was significantly correlated with baseline self-efficacy, but not with post-intervention self-efficacy. However, the latter was significantly and positively correlated with communication skill competence. The participants who had higher post-intervention self-efficacy in conducting clinical handovers also performed better in the communication skill competence assessment.

### Between-subjects effects

The communication skill competence assessment scores and self-perceived self-efficacy in conducting clinical handovers of the participants from both groups at 2 weeks after the intervention are presented in Table 3. The baseline and post-intervention self-efficacy were considered as covariates, while the other variables that were correlated with them were excluded to prevent collinearity. The participants in the experimental group reported significantly higher mean scores in the skill competence assessment than those in the waitlist control group; (F(1, 86) = 113.18, *p* < .001, η^2^ = .57).

**Table 3:** Between-Subjects Effects of the Clinical Handover Training Course on the Communication Skill Competence and Self-Efficacy at two weeks after the intervention (*N* = 89)

		Control (n = 41)	Experimental (n = 48)	ANCOVA			
DV	COV	M (SD)	M (SD)	***F***-value	P	Partial η^**2**^	Power
OSCE	Post SE	31.00 (4.99)	46.02 (7.32)	F (1,86) = 113.18	< 0.001	0.568	1.00
Post SE	Baseline SE	51.54 (12.10)	60.94 (15.11)	F (1,86) = 20.83	< 0.001	0.195	0.995

*DV* Dependent variable, *COV* Covariate, *OSCE* Skill competence scores, *SE* Self-efficacy

95% confidence interval

The mean self-efficacy scores were significantly higher in the experimental group than in the waitlist control group after controlling for the mean self-efficacy scores on the pretest; ((F1, 86) = 20.83, *p* < .001, η^2^ = .20).

## Discussion

Communication skills and self-efficacy are essential components of handing over the responsibility of patient care to other healthcare professionals. The overall results demonstrate the efficacy of the proposed intervention in enhancing the clinical handover practice of nursing students.

Clinicians’ performance in clinical handovers is influenced by many factors; clinical experience is an essential one of them [41, 42]. In fact, experienced nurses usually perform better in receiving and conducting clinical handover [43]. The results of this study also showed that the students’ previous experience of observing and performing clinical handovers was significantly correlated with their baseline self-efficacy, but not the post-intervention self-efficacy. These findings indicated that although previous experience of clinical handover practice was important, the clinical handover training had a more impactful role in enhancing the students’ self-efficacy in conducting clinical handover. Moreover, despite the participants in both groups showed a significant improvement in self-efficacy, the experimental group showing a markedly higher improvement. Besides, students who received blended clinical handover training achieved significantly higher mean scores in communication skill competence assessments than those who received only a face-to-face training workshop These mean the intervention that the experimental group received is more effective than the control group. In the current Hong Kong nursing curriculum, a face-to-face training workshop is the most common teaching strategy used to prepare nursing students for clinical handover. This study demonstrates that an online module provided an additional effect to the existing face-to-face training workshop. Previous studies also suggested that combining large class teaching and online activities facilitates clinical handover trainings [44, 45]. These findings demonstrate the importance of incorporating a blended learning approach in the curriculum; thus, a combination of a face-to-face training workshop with an online module can efficiently teach nursing students about clinical handovers. The online module with scenario-based exercises encouraged the students to make use of the SBAR technique when conducting clinical handovers and provided more opportunities for them to practice these skills, which consequently improved their self-efficacy and communication skill competence. Noh and Lee’s research [46] also showed that participants’ awareness of using SBAR technique and communication self-efficacy increased gradually after attending lectures and participating in scenario-based role-plays. In addition, Jeong and Sook’s research [47] found that after receiving communication training program, nursing students not only improved their communicative competence but also the interpersonal relations. These findings were consistent with previous studies conducted on medical interns and postgraduate residents that demonstrated the efficacy of using online modules to improve their self-efficacy in conducting clinical handovers [30, 48].

### Limitations

This study had some limitations. First, the students who chose to participate might have had higher motivation and willingness to learn the skill, making the intervention more effective for them. Second, the outcomes were measured at the baseline and immediately post-intervention, leaving the long-term effects of the intervention unknown. Third, the study was conducted in a single centre, limiting the generalisability of its findings. Finally, the SBAR communication tool was used to measure the communication skill competence, which aimed to examine the students’ skill performance in adopting the SBAR technique for clinical handovers. This implied that the communication skill competence was not assessed at the beginning of the study as the students had not learned the technique by then. In future studies, pretest data on communication skill competence should be collected immediately after the face-to-face training workshop. Conducting the pretest after the training workshop is an alternative way of collecting data as all of the participants would by then have the basic knowledge and skills required to adopt the SBAR technique for clinical handovers. Comparing their performances after the face-to-face training workshop and after the experimental group completes the online learning will help determine whether the online module has an additional effect on the communication skill competence of the participants in the experimental group.

### Implications

The results of this study suggest a need for more training for undergraduate nursing students. The acquisition of knowledge and ample practice of skills will raise their self-efficacy and improve their skill competence. To achieve this goal, a blended learning approach can be used to teach nursing students about clinical handovers. A BLP provides plenty of learning opportunities in a flexible manner for students to practice their skills. Using a blended learning approach for clinical handovers is an innovative teaching strategy that can improve the skill competence of nursing students, enhance the quality of nursing care and promote patient safety [49]. Moreover, the online component of the training programme is particularly advantageous during pandemics, when face-to-face teaching is not feasible. It allows students to learn in safe environments while practicing social distancing. In addition, the communication skill competence assessment conducted in this study was developed based on the Objective Structured Clinical Examination (OSCE). The results of this study demonstrate the utility of the OSCE, which can be incorporated in future studies to objectively assess the actual performance of the participants’ handover reporting skills. Most importantly, the results of this study can be used to optimise the content of training programmes for clinical handover in the nursing curriculum.

## Conclusion

This study supports the efficacy of using a blended learning approach to enhance the communication skill competence and self-efficacy of nursing students in conducting clinical handovers. Based on the findings of this study, it is recommended that nurse educators should incorporate a blended learning approach into the nursing curriculum to optimise the content of training programmes for clinical handover with the aim of preparing nursing students for their future roles and responsibilities after graduation.

## Supplementary Information

**Additional file 1: Supplement 1.** Application of Madeline Hunter’s model to the clinical handover training programme.

## Data Availability

The datasets generated and/or analysed during the current study are not publicly available due to privacy and ethical issue but are available from the corresponding author on reasonable request.
