# Supplementary material for: Efficacy of a blended learning programme in enhancing the communication skill competence and self-efficacy of nursing students in conducting clinical handovers: a randomised controlled trial
Source: BMC Med Educ. 2022 Apr 13;22:275. doi: 10.1186/s12909-022-03361-3 (PMC9009000; doi:10.1186/s12909-022-03361-3)
Supplement: Supplementary file 1 — Additional file 1: Supplement 1. Application of Madeline Hunter’s model to the clinical handover training programme. [file 12909_2022_3361_MOESM1_ESM.docx]

Supplement 1

Application of Madeline Hunter’s model to the clinical handover training programme

| **Step** | **Main component** | **Timing** |
| --- | --- | --- |
| 1. **Anticipatory set:** create a desire to learn | - Sharing an incident report that a related to ineffective clinical handover. - Discuss the common barriers to clinical handovers. | 15 mins |
| 1. **Standards:** provide the learning outcomes | - Provide and explain the learning outcomes. | 5 mins |
| 1. **Teaching & modelling:** provide a model of what is expected as the end product of the learning | - Introduce criteria of effective clinical handover. - The function of clinical handover. - The benefits of verbal clinical handover. - The responsibilities when giving/receiving clinical handovers - Introduction of SBAR technique. - The usages of SBAR in   - doctor-nurse communication   - changes in patient health status   - between shifts   with demonstration of a case scenario. | 40 mins |
| 1. **Guided practice:** Students work on activities | - Two scenarios of doctor-nurse communication and handover between shifts are given to practice formulating SBAR handover. | 30 mins |
| 1. **Check for understanding:** invite students to return demonstrate and encourage raise questions | - Teacher gives feedback on their handover reports. - Questions and open discussion are encouraged. - Face-to-face workshop closure | 30 mins |
| 1. **Independent practice:** repeat the practice to reinforce the learning | - Participants in the experimental group access the 2-week online module to practice the handover skills with some scenario-based exercises. | 2 weeks |
| 1. **Closure:** Provide acknowledgement to students | - Post-intervention assessment   - OSCE for communication skill competence   - VAS for self-efficacy   - Questionnaire for students’ satisfaction | 30 mins (for OSCE)  15 mins |
